# Vascular Aging in the Invertebrate Chordate, *Botryllus schlosseri*

**DOI:** 10.3389/fmolb.2021.626827

**Published:** 2021-04-08

**Authors:** Delany Rodriguez, Daryl A. Taketa, Roopa Madhu, Susannah Kassmer, Dinah Loerke, Megan T. Valentine, Anthony W. De Tomaso

**Affiliations:** ^1^Department of Molecular, Cellular and Developmental Biology, University of California, Santa Barbara, Santa Barbara, CA, United States; ^2^Department of Physics and Astronomy, University of Denver, Denver, CO, United States; ^3^Department of Mechanical Engineering, University of California, Santa Barbara, Santa Barbara, CA, United States

**Keywords:** vascular cells, *Botryllus*, branching, heritable, cell-morphology, aging, blood flow

## Abstract

Vascular diseases affect over 1 billion people worldwide and are highly prevalent among the elderly, due to a progressive deterioration of the structure of vascular cells. Most of our understanding of these age-related cellular changes comes from *in vitro* studies on human cell lines. Further studies of the mechanisms underlying vascular aging *in vivo* are needed to provide insight into the pathobiology of age-associated vascular diseases, but are difficult to carry out on vertebrate model organisms. We are studying the effects of aging on the vasculature of the invertebrate chordate, *Botryllus schlosseri*. This extracorporeal vascular network of *Botryllus* is transparent and particularly amenable to imaging and manipulation. Here we use a combination of transcriptomics, immunostaining and live-imaging, as well as *in vivo* pharmacological treatments and regeneration assays to show that morphological, transcriptional, and functional age-associated changes within vascular cells are key hallmarks of aging in *B. schlosseri*, and occur independent of genotype. We show that age-associated changes in the cytoskeleton and the extracellular matrix reshape vascular cells into a flattened and elongated form and there are major changes in the structure of the basement membrane over time. The vessels narrow, reducing blood flow, and become less responsive to stimuli inducing vascular regression. The extracorporeal vasculature is highly regenerative following injury, and while age does not affect the regeneration potential, newly regenerated vascular cells maintain the same aged phenotype, suggesting that aging of the vasculature is a result of heritable epigenetic changes.

## Introduction

While vascular tissues play a major role in organismal aging, they are one of the most challenging to study *in vivo*: the large and continuous network branches to distinct vascular beds and interacts with myriad tissues and organs. Moreover, vascular tissues are found deep within the animal and are largely inaccessible to optical imaging and experimental manipulation. Because of these challenges many studies are performed *in vitro* using cell cultures that allow for greater control of the environmental conditions and improve reproducibility. However, these studies fail to reproduce physiological conditions such as the interaction of vascular cells with the basement membrane, fluid shear stress from blood flow, and signals from surrounding tissues, amongst others (Weinstein, 2002; Kinlay et al., 2016; Serra et al., 2018; Song et al., 2018; Cochrane et al., 2019). Studying aging *in vivo* also requires that the cell- and tissue-level dynamic changes be followed over the lifespan of an organism, adding another level of complexity (Donato et al., 2015, 2018; Xu et al., 2017; Bersini et al., 2020). Here we address these challenges using a novel model system which allows us to study vascular aging *in vivo*: the extracorporeal vasculature of the invertebrate chordate, *Botryllus schlosseri*.

As described in detail below, *Botryllus* has a large, extracorporeal vascular network that is an excellent model for vascular aging due to its size, accessibility and transparency (Gasparini et al., 2007, 2008, 2014; Tiozzo et al., 2008; Rodriguez et al., 2019), In addition, the *Botryllus* vasculature is highly regenerative. Angiogenesis occurs during the whole asexual cycle of the organism, and can also be stimulated by ablation of the vascular bed (Gasparini et al., 2007, 2008; Tiozzo et al., 2008). And similar to vertebrates, any region of the vasculature can be induced to grow: there are no vascular progenitors and all cells appear to have the potential to re-enter the cell cycle (Tiozzo et al., 2008; Braden et al., 2014; McDonald et al., 2018). *Botryllus* has been used as a model to study different aspects of aging such as the impact of reproduction on lifespan, non-random senescence, and rejuvenescence, among others (Brunetti and Copello, 1978; Rinkevich et al., 1992; Chadwick-Furman and Weissman, 1995; Voskoboynik et al., 2002; Munday et al., 2015; Rinkevich, 2017; Ben-Hamo et al., 2018).

*Botryllus* is an ascidian, a group of marine invertebrate chordates that are considered the closest living ancestors of vertebrates (Delsuc et al., 2006; Kocot et al., 2018). Upon internal fertilization, the *Botryllus* embryo develops into a chordate tadpole, that hatches out of the mother and in the next 36–48 h undergoes a dramatic metamorphosis to the sessile adult body plan, called a zooid (Sabbadin, 1955; Manni et al., 2007; Gasparini et al., 2015; Rodriguez et al., 2016). Zooids have a complex anatomy, including a gastrointestinal tract, central and peripheral nervous system, a heart and complex hematopoietic system. In addition, *Botryllus* is colonial, and metamorphosis is followed by a lifelong budding asexual reproductive process: each week, when maintained at a water temperature of 18–19°C, the adult zooid develops up to four primary buds that grow from the body wall. After the primary bud(s) complete development, the adult zooid dies and is resorbed by blood-borne phagocytic cells allowing the primary buds to replace the old zooids and become the new adult zooids (Manni et al., 2007, 2019). In maintenance, each dying zooid is replaced by one primary bud, whereas in expansion, a dying zooid is replaced by more than one primary bud thus increasing colony size.

**Figure 1:**
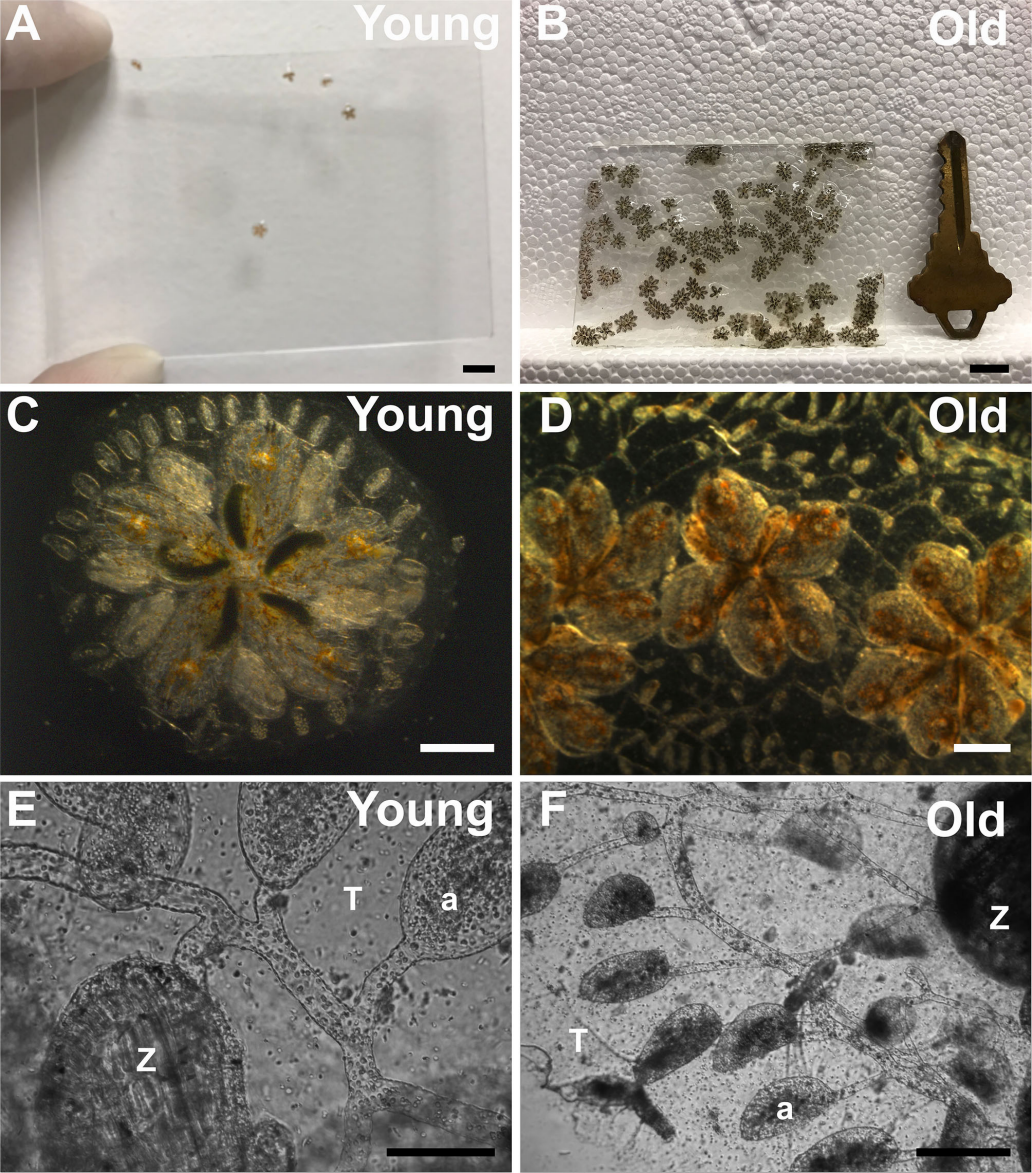
Growth, expansion, and senescence of *Botryllus* colonies. **(A)** Young and **(B)** old *Botryllus* colonies on 7.6 × 5 cm glass slide. **(C)** Close up of a single young and **(D)** old *Botryllus* colony. **(E)** Close up to the extracorporeal vasculature of a young and **(F)** old *Botryllus* colony including zooid (Z) and ampullae (a) and tunic (T). Scale bars: **(A,B)** = 10 mm, **(C,D)** = 1 mm, **(E,F)** = 50 μm.

All zooids are interconnected by an extracorporeal vasculature and share a common blood supply (Figures 1A–D). The vasculature forms a large network of vessels that extends underneath and surrounds the outside of the zooids. At the periphery of the colony, the vessels terminate in finger shaped protrusions called ampullae (Figures 1E,F). Finally, the zooids and vasculature are embedded in a clear, extracellular matrix (ECM) called the tunic (Figures 1E,F).

The basoapical polarity of *Botryllus* vessels is reversed in comparison to vertebrates, and they consist of a flat epithelium of ectodermally-derived cells with the basal lamina facing the lumen and the apical side facing the tunic (Gasparini et al., 2007). The vasculature is very dynamic, and expands as the number of zooids increases. In addition, the entire vascular bed can fully regenerate following surgical ablation (Gasparini et al., 2008; Tiozzo et al., 2008; Braden et al., 2014). Finally, angiogenesis is dependent on conserved growth factors such as VEGF, angiopoietins, and FGF-2 (Gasparini et al., 2007, 2008; Tiozzo et al., 2008).

One of the most interesting aspects of aging in *Botryllus* is that a single genotype (colony) has a lifespan, which ranges from months to years (with an average of 1 year in the wild and >3 years in laboratory conditions) (Grosberg, 1981, 1988; Chadwick-Furman and Weissman, 1995; Lauzon et al., 2000; Munday et al., 2015). However, the zooids are transient structures with a lifespan of only 3 weeks (at a water temperature of 18–19°C), during which they feed, asexually and sexually reproduce; then die and are resorbed by the colony (Lauzon et al., 1992; Manni et al., 2007, 2019). Thus, during its life history a colony experiences constant turnover of zooids, and in contrast to long-lived tissues such as the vasculature, tunic, blood circulating germline stem cells among others. Therefore, *Botryllus* allows the study of dynamic age-related changes to the vascular tissue over its relatively short lifespan and provides unique opportunities for non-invasive observation and experimental manipulation of this tissue *in vivo*.

Prior studies of aging in *Botryllus* have focused on qualitative descriptions of genotypic death, which progresses through distinct morphological stages (Brunetti, 1974; Rinkevich et al., 1992; Chadwick-Furman and Weissman, 1995). The first noticeable characteristic of an older genotype is narrowing of the vessel diameters and a decrease in blood flow. Concurrently, asexual reproduction slows down, the number of zooids is either maintained or reduced causing colonies to stop expanding, and the formation of new vasculature is halted. Next, the zooids shrink and become heavily pigmented, and the bodies shrink, their spatial organization in the colony becomes disorganized, and they lose their vascular connections with neighbors. Finally, death occurs simultaneously throughout the colony: all zooid are resorbed, and there is a complete cessation of blood circulation.

In this work, we leverage the transparency and accessibility of the extracorporeal vascular network of *Botryllus* for quantitative analysis of aging phenomena. Using a combination of transcriptomics, immunostaining and live-imaging, as well as *in vivo* pharmacological treatments and regeneration assays, we address several important questions regarding vascular aging in *Botryllus*: What are the key genes driving aging in *Botryllus* colonies? What age-related changes occur at the cellular level in vascular cells? What is the effect of age-dependent vessel constriction on vascular cells and blood flow? Does aging have an effect on vascular dynamics (i.e., regression and/or regeneration)?

Our results show that the vasculature of *Botryllus* undergoes a number of age-related transcriptional changes in genes encoding regulators of cytoskeletal and ECM properties that are consistent among genotypes. Specifically, aged colonies express lower levels of both ECM and cytoskeleton genes, which correlates with altered cytoskeletal organization and cell shape. In turn, these ECM and cytoskeleton alterations correlate to the functional decline of aged vasculature, including changes in vessel diameter and blood flow. In a previous study, we found that pharmacological inhibition of collagen crosslinking enzymes induced global vascular regression in young animals (Rodriguez et al., 2017; Madhu et al., 2020); here we find that this response is age-dependent, and significantly reduced in older colonies. Finally, we found that regeneration of ablated vasculature does not depend on age. However, the age-dependent changes in the architecture of the ECM and the cytoskeleton are retained in the regenerated cells, suggesting that age-related changes are permanent and heritable. The observed correlation between age-related morphological changes and functional changes in an *in vivo* model of vascular aging demonstrates the value of *Botryllus* as a model providing insight into the biology of vascular aging.

## Materials and Methods

### Animals

Multiple wild-type *Botryllus schlosseri* (herein called “*Botryllus*”*)* colonies were collected from the yacht harbor in Santa Barbara, CA, spawned and cultured in laboratory conditions at 18–20°C according to established protocols by Boyd et al. (1986). Animals are reared in 5 L tanks and fed daily with live algae in suspension and food is not limiting (Rodriguez et al., 2014). Colonies were staged-matched based on blastogenetic stage cycle B2 according to Lauzon et al. (2002) and all experiments were performed at this stage.

### Blood Vessel Diameter and Blood Flow Analysis

**Figure 2:**
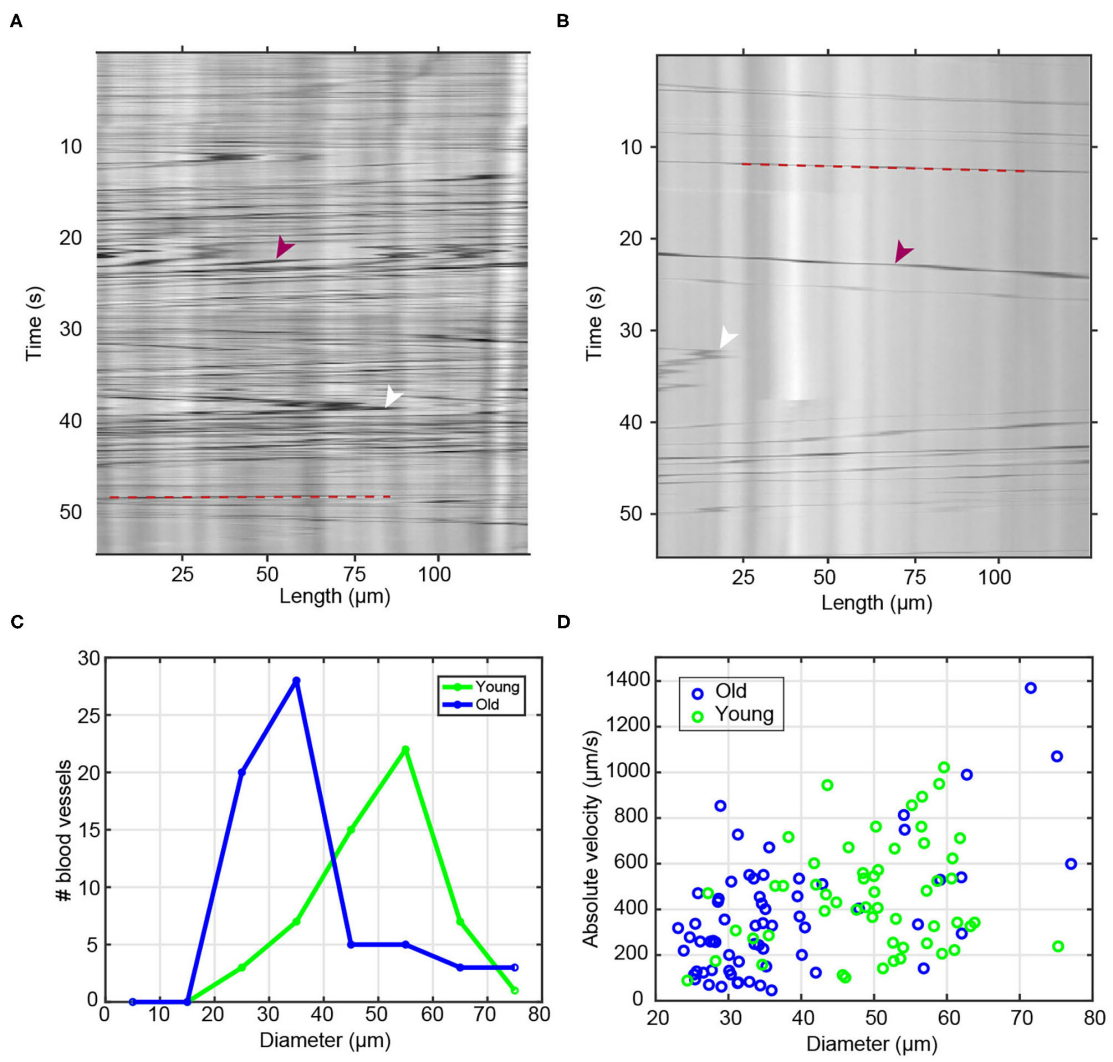
Blood flow and diameter analysis of young and old vessels. **(A,B)** Raw sample kymographs of blood vessels from **(A)** young and **(B)** old animals. Red arrowheads show the high contrast objects/cells and white arrowheads point toward reversals of blood flow direction. Red dashed lines highlight the trajectory of a blood cell. **(C)** Histogram of measured blood vessel diameter in young (*n* = 55) and old (*n* = 64) animals. **(D)** Scatter plot of measured maximum flow velocity as a function of blood vessel diameter.

To record time-lapses of blood flow we used an inverted Olympus IX71 (Tokyo, Japan) with a 20x objective and acquired recordings at 8.55 frames/second for 1 min for each blood vessel. We measured a total of *n* = 55 blood vessels from *k* = 8 young colonies (4 months), and a total of *n* = 64 blood vessels from *k* = 18 old colonies (3 years). Image analysis software Fiji was used for kymograph generation, using continuous (non-branched) sections of the vessel for blood flow analysis. In the minimal projection image of the vessel, blood vessel diameter was determined by manually drawing a line across the blood vessel (Supplementary Figure 1A). In the same image, a line segment region of interest (ROI) was manually drawn along the center line of a non-branched section of the vessel (Supplementary Figure 1A). This ROI with a thickness of ~3 μm was then used to generate a kymograph using the tool *Multi Kymograph*. The kymographs (Figure 2A) were stored as .tif files and imported into *MATLAB* (Mathworks) for quantification of blood flow. The kymographs are shown with time on the *y*-axis and axial position (along the blood vessel) on the *x*-axis. Vertical stripes in the kymographs are caused by immobile objects or intensity variation along the vessel, and are removed through subtraction of the variable background intensity, specifically by subtracting a rolling average (over 20 frames) from the raw kymograph. This background-subtracted image is then normalized by subtracting the mean and dividing by standard deviation before further analysis. Blood flow velocities were measured using a 2-dimensional autocorrelation analysis on the normalized kymograph: Specifically, for each time point, the analysis extracts a local section of the kymograph of length *t* = 3 frames, and finds the distance shift Δ*x* that produces the maximum 2D autocorrelation magnitude for Δ*t* = 1 frame. Maximum flow velocity for this time point is then calculated as *v* = Δ*x*/Δ*t*; flow velocities in [pixels/frame] are converted to [μm/sec] using the following measured conversion factors: 0.313 μm/pixel and 0.177 s/frame.

In those regions of the kymograph where few or no high-contrast particles are present, autocorrelation analysis is dominated by noise and produces no meaningful results; these low-contrast regions are associated with low absolute magnitudes of the autocorrelation function. Thus, to constrain the analysis to the regions of the kymograph with high-contrast particles, the flow velocity analysis is constrained to the regions with autocorrelation intensities >0.8.

### Transcriptome Analysis of Young and Old *Botryllus* Colonies

To explore only the genes involved in aging, mRNA seq analysis was performed at stage B2 of the blastogenic cycle as described by bud morphogenesis based on *in vivo* and histological features (Lauzon et al., 2000) on a total of 3 young (4 months) samples consisting of 3 systems per sample (genotypes: SB825, SMH, and SMGO) and 3 old (3 years old) samples consisting of 3 systems per sample (genotypes: SB825, SMH, and SMGO).

Total RNA was isolated from whole colonies using the Nucleo-Spin RNA II kit (Macherey-Nagel, Bethlehem, PA, USA). Libraries were prepared and sequenced at the University of Southern California Epigenome Center using kits from Illumina following the manufacturers' instructions. Paired-end libraries were generated for each sample and sequenced with an Illumina Hi-Seq 2000. Following RNASeq each paired end library was immediately checked for quality control using the software FastQC with an average score of 28 across all bases (Illumina 1.5 encoding). Using FASTQ Trimmer we removed the adapters of the sequences by removing the first 12 bases of each read. All reads pairs passing quality control were mapped using BOWTIE (2012 version 0.12.7) (Langmead et al., 2009) to the public EST database Bot_asmb assembly (04.05.2011, A. Gracey) (http://octopus.obs-vlfr.fr/public/botryllus/blast_botryllus.php). Bot_asmb assembly (04.05.2011, A. Gracey), consisting of 50,107 contigs. The number of reads mapping to each EST was obtained with Sam2Counts (Samtools version 0.1.18) and differential expression analysis was performed with DESeq 1.10.1 using triplicates for the analysis and a false discovery rate of 10 % (Anders and Huber, 2010). We manually excluded genes known to be involved in fertility and sexual maturation based on our previous studies (Rodriguez et al., 2014, 2016) including *otoancorin, P-selectin*, β-*estradiol, tetraspanin-8, testis-specific serine/threonine-protein kinase 1 and 2*, s*permatogenesis-associated protein 6, low density lipoprotein*, and *tenascin* among others. Our spatial studies of these genes showed that they are expressed by gonads or germline precursors and not in vascular tissue (Rodriguez et al., 2014).

### Gene Ontology Analysis

The list of human homologs of differentially expressed *Botryllus* contigs was submitted to GeneCodis 3, an on-line modular enrichment tool (Carmona-Saez et al., 2007; Nogales-Cadenas et al., 2009; Tabas-Madrid et al., 2012). For this analysis, the following annotations for GO Biological Process were selected. The statistical parameters for these analyses were as follows: First, for co-occurrence analyses of annotations, a minimum support of 3 genes was required. Second, the statistical method to compute *p*-values was the hypergeometric test. Finally, to correct *p*-values for multiple hypothesis testing, false discovery rate estimation was utilized. Of particular interest to this study were processes and pathways with a known role in aging.

### Immunostaining of Young and Old *Botryllus* Colonies

Briefly, colonies were anesthetized with 800 nM Tricane (TCI, Tokyo T0941) for 10 min at room temperature. Whole colonies were fixed with 4% Paraformaldehyde in 0.1M MOPS, 0.5M NaCl for 3 h at room temperature, then washed and permeabilized with PBS-Tween (or Triton X-100) at 0.05%. Samples were blocked with 2% BSA for 2 h at room temperature, incubated with primary antibody at 4**°**C for 48 h, and then washed with PBS. Next, samples were incubated with secondary antibody at 4**°**C for 48 h and then washed with PBS. Antibodies were used at the following dilutions: Anti Pan-Cadherin (Cell Signaling Technology, Danvers, MA, Cat. No. 4068, 1:100). Monoclonal antibodies anti α-Tubulin (hybridoma bank 1:250, product number 12g10, developed by Frankel, J and Nelsen, E. M), and anti- Collagen-II (hybridoma bank 1.5:100, product number II-II6B3, developed by Linsenmayer, T. F.) were obtained from the Developmental Studies Hybridoma Bank, created by the NICHD of the NIH and maintained at the University of Iowa, Department of Biology, Iowa City, IA 52242. Antibodies were detected using a secondary antibody (either anti- mouse or anti-rabbit specific to each primary antibody) conjugated to Alexa Fluor 488 detected with an excitation/emission 499/520 (Thermo Fisher Scientific, Waltham, MA Cat. No. A11008, A11012). Samples were counterstained with Rhodamine Phalloidin to stain F-Actin by incubation overnight with a dilution 1:100 (Thermo Fisher Scientific, Waltham, MA Cat. No. R415); simultaneously DNA staining was performed by incubation overnight with Hoechst 33342 with a dilution of 1:1000 (Thermo Fisher Scientific, Waltham, MA Cat. No. H3570). Excess dyes were washed 3x with PBS. Samples with Phalloidin were detected with an excitation/emission 540/565 and Hoechst 33342 was detected with an excitation/emission 350/461. In all cases, samples were imaged on an Olympus FluoView 1000 spectral confocal microscope (Tokyo, Japan), using a 40x oil immersion objective. To quantify the fluorescence signal on immunolabeled samples with anti- Collagen-II (detected with a secondary antibody anti-mouse Alexa 488), we acquired confocal *z*-stacks micrographs of the vascular bed. The *z*-stacks were rendered using ImageJ and then using the polygonal selection tool regions of interest (ROI) were selected where positive signal was detected, and the intensity of the signal was calculated by measuring the mean intensity of the vessel using FIJI (Schindelin et al., 2012). A second ROI of background only was also acquired for each micrograph. To calculate the corrected total fluorescence (CTF), we used the following equation: CTF = Integrated intensity—(ROI × Mean fluorescence of background). All samples were normalized and compared with the controls (*n* = 6); the standard deviation of the mean was calculated and two-tailed *t*-test was used to obtain statistical significance using Microsoft Excel for each data set.

### Morphometrics of Vascular Cells

Samples that were immunolabeled with Anti-Pan Cadherin were imaged with a confocal microscope [Olympus FluoView 1000spectral confocal microscope (Tokyo, Japan)] and *z*-stacks were collected with a step size of 2 μm. The *z*-stacks were then rendered into a single 2D image using ImageJ, and analyzed using the built-in Circularity measurement tool which returns a measure of the shape, using the formula *circularity* = *4*π*(area/perimeter*^∧^*2)*, where a circularity value of 1 indicates a perfect circle. Using the same rendered *z*-stacks, the area of the vascular cells was measured using ImageJ using the built-in Area measurement tool. The area and circularity values for each rendered micrograph were then exported to Excel where the average value and standard deviation were calculated and a two-tailed *t*-test used to calculate the significance of the difference between compared values.

### Regeneration Assay of Blood Vessels (Ampullectomy)

To study vascular regeneration in both young and old colonies, we followed the procedure described by Gasparini et al. (2008), Tiozzo et al. (2008), and Braden et al. (2014). Briefly, the extracorporeal vasculature including ampullae were surgically removed, leaving only the zooids and the vasculature that interconnects them and is thus not accessible for surgery. Animals were allowed to recover in our mariculture facility with flowing seawater and food for the following 6 days. Bright field images were taken before and immediately after surgery and again 6 days later to show recovery. Control and regenerated colonies were then fixed, immunostained with Anti Pan-Cadherin and imaged, and those images analyzed as described in the previous sections.

### Drug Treatments

LOX1 inhibition was accomplished by using the specific small molecule ß-aminopropionitrile (BAPN) (MP Biomedicals, Santa Ana, California, USA. Cat. No. 150105) diluted in 500 mL of filtered seawater to the concentration of 400 μM. *Botryllus* colonies were allowed to soak in either a 0.5-μm filtered seawater (FSW) control with the same amount of molecular biology grade water (same carrier as BAPN) or the BAPN-containing solution for 16 h at 18–20°C prior to measurements of vascular regression (*n* = 15, 3–6 months old). For time-lapse imaging of live tissues, BSA-Alexa Fluor 594 (Thermo Fisher Scientific Waltham, MA 1 mg/ml in PBS) staining was performed by microinjection directly into the vasculature and allowed to incubate for 24 h before imaging. Live imaging of young and old colonies were treated with BAPN and recorded using a motorized fluorescence stereomicroscope MZ16FA (Leica, Germany).

To calculate regression, the size of the vascular bed (*S*_*vb*_) was calculated by subtracting the perimeter of the border of the bodies (*p*_*b*_) from the perimeter of the border of the ampullae (*p*_*a*_), *S*_*vb*_ = *p*_*a*_–*p*_*b*_. Each boundary was identified manually using the polygonal selection tool of ImageJ and then the perimeter of the boundary measured using the built-in Perimeter tool for both *p*_*b*_ and *p*_*a*_, and the results were exported to Excel. The percentage of regression was calculated as: (*S*_*vb, ctrl*_−*S*_*vb, regres*_/*S*_*vb, ctrl*_) x 100, where *S*_*vb, ctrl*_ is the vessel bed size before drug treatment, and *S*_*vb, regres*_ is the vessel bed size after drug treatment. A paired two tailed Student's *t*-test was applied the mean value of the percentage of regression using Excel to determine the significance of comparisons between young and old samples.

### RT-PCR, Cloning, and Quantitative PCR

Surgeries were performed to remove all individuals from the colonies and specifically assess gene expression within the vascular tissue. First mRNA was isolated from vascular tissue by using the NEB Magnetic Bead Isolation kit. cDNA was made using Superscript II from Invitrogen. For RT-PCR, we used Clontech Advantage following the manufacturers′ recommendations for PCR conditions.

Quantitative PCR (qPCR) analysis was performed as described (Rodriguez et al., 2014; Kassmer et al., 2015). Briefly, qPCR was performed using a LightCycler 480 II (Roche) and LightCycler DNA Master SYBR Green I detection (South San Francisco, CA Roche, 12015099001). The thermocycling cycle was as follows: 5 min at 95**°**C, 45 cycles of 95**°**C for 10 s, 57**°**C for 10 s, and 72**°**C for 10 s. All gene expression data was normalized to *elongation factor 1*-α (*EF1-*α) as a reference house-keeping gene and reported as relative expression using the 2^−ΔΔCt^ method. Three biological and three technical replicates were used for each gene. Results are shown as average of relative expression ratio (fold change) and normalize to young vascular tissue. Primers for qPCR are (5′-3′ forward and reverse): Actin: TCTGTTGACGGAAGCTCCAC, TCGTAGATTGGGACGGTGTG, Tubulin: TTGAGTTCGCCATCTACCCG, CCGACGAGACGGTTCAAGTT, Collagen-1: GTTTCCAGTCGCAATCTCACG, GGTGAACTACAAAGCTGCCG, Collagen-2: CAGTCACTCCGACAGCCAAT, TAACTGGGGATACCCGGACC, Lysyl oxidase: ATGGGGAACAGTCTGCGATG, CAGCTGTTTTTCGGGCAGTC, Tenascin-r: TCGACACTTCCATCCGTTCG, TTGGCATTAACGCCGCAAAA.

## Results

### Growth, Expansion, Branching, and Aging of the Extracorporeal Vasculature

Taking advantage of the large, long-lived, transparent and extracorporeal vascular bed of *Botryllus*, we characterized age-related changes to this tissue among multiple wild-type genotypes at stage B2 of the blastogenetic cycle. The first obvious hallmark of aging is the constriction of vessel diameter which as a consequence slows blood flow (Brunetti, 1974; Rinkevich et al., 1992; Chadwick-Furman and Weissman, 1995; Lauzon et al., 2000; Munday et al., 2015; Voskoboynik and Weissman, 2015). To quantitatively study age-related changes in blood flow, we used time-lapse imaging to record the circulating blood of both young and old colonies recording *n* = 55 blood vessels for young colonies (4 months) (Supplementary Video 1) and *n* = 64 blood vessels for old colonies (3 years) (Supplementary Video 2). As shown in the representative kymographs of young and old colonies (Figures 2A,B, respectively), we systematically observed a much higher density of high-contrast particles in young animals (Figure 2A) as compared to old ones (Figure 2B). We also measured the age-dependent changes in blood vessel diameter. We observed that young blood vessels tend to be significantly wider than old ones, with young colonies having a typical blood vessel diameter (mean ± SD) of 49.6 ± 10.4 μm, and old colonies having diameters of 37.3 ± 13.1 μm (significant at *p* = 1.50 × 10^−7^, two-sample *t*-test) (Figure 2C).

Next we calculated the flow velocity, and found that the observed velocity increased systematically with blood vessel diameter (Figure 2D) within both the young and the old populations–a behavior that is qualitatively consistent with the flow dynamics with branched vascular networks in other organisms (including humans), where flow velocity is highest in the aorta and lowest in the capillary bed. We confirmed quantitatively that the observed correlation between velocity and vessel diameter was in good agreement with Murray's Law (Sherman, 1981). A data fit of the measured volumetric flow rate against vessel diameter (Supplementary Figure 1F) yields a power law with an exponent (also called a “branching exponent”) of approximately *n* = 3.05; this value is close to the theoretical value of *n* = 3 predicted by Murray's Law (Murray, 1926), and comparable to experimentally measured branching exponents in other biological systems, e.g., an exponent of *n* ~ 2.8 in human retinal vasculature (Riva et al., 1985).

Once we account for the dependence of flow velocity on vessel diameter, we do not observe significant velocity differences between young vs. old vessels. When we specifically compare vessels of similar diameter (e.g., those in the range of 25–35 μm, or those in the range of 55–65 μm) between the two age categories, we observed similar flow velocities between young and old. For example, for vessels in the diameter range of 25–35 μm, we measured flow velocities of (mean ± std) *v* = 276 ± 125 μm/s in young and *v* = 290 ± 198 μm/s in old vessels (Supplementary Figure 1E). These data suggest that within our level of experimental resolution, any differences in measured flow velocities between young and old blood vessels likely arise from the different distribution of vessel diameters in young vs. old vascular beds, as opposed to gross differences in blood pressure or in the mechanical properties of the vessels between young and old animals.

### Identification of Key Genes Expressed During Aging of *Botryllus* Colonies

To determine aging-related molecular changes, we profiled the transcriptomes of young and old wild-type colonies. We used mRNA-Seq to comprehensively search for genes associated with aging by comparing differentially expressed genes between young (4 months) and old (3 years) individuals. The sequences were mapped to our publicly available *Botryllus schlosseri* EST database Bot_assmb assembly (04.05.2011, A. Gracey) (http://octopus.obs-vlfr.fr/public/botryllus/blast_botryllus.php). To identify putative homologs of the EST in our database we used the translated basic local alignment search tool (BLASTx) using the non-redundant human protein database (NCBI version 4/25/13) as well as the non-redundant protein database for *Ciona sp* (NCBI version5/12/13 taxid: 7719) (an E-value of 1.0 x 10-4 was chosen as a cutoff for a homolog for the purpose of this study). Using differential expression analysis (using 3 genotypes and 3 biological replicates for each genotype) we found 7,695 differentially expressed genes in aging *Botryllus* colonies, with 3142 human homologs (Table 1, Supplementary Tables 1, 2) (the data supporting the results of this article have been deposited in NCBI's Gene Expression Omnibus and are accessible through GEO Series record number GSE115267 https://www.ncbi.nlm.nih.gov/geo/query/acc.cgi?acc=GSE115267). Our initial analysis focused on genes with the highest fold change and these were organized by gene family; we excluded genes related to fertility and sexual maturation. The top ten differentially expressed genes are shown in Table 2 (see Supplementary Table 1 for all differentially expressed genes and fold changes). These genes relate to the actin and tubulin cytoskeleton, apoptosis (caspase 2), cell division (cell division cycle protein 23), and extracellular matrix (collagen).

**Table 1:** Number of differentially expressed EST's between young and old colonies and number of those EST's with human homologs and gene ontology annotations.

**Number of differentially expressed genes (10% FDR)**	**Number of human homologs**	**Gene ontology annotations**
7,695	3,142	350

**Table 2:** Top ten differentially expressed genes not involved in fertility.

**Human homolog**	***Ciona* homolog**	**Fold change**	**Adjusted *p*-value**	**Contig ID**
Actin cytoplasmic	Actin	4.88	0.00837176	CAP3_round1_contig_6729
Actin related protein 2/3	Actin related protein 2/3	3.52	0.00599977	Bot_rep_c48230
Caspase 2	Caspase 2-like	3.43	0.08624975	Bot_c10337
Cell division cycle protein 23	Cell division cycle protein 23	2.73	0.01481089	Bot_rep_c37856
Collagen α 1 (II) chain	Fibrillar Collagen precursor	2.12	0.08968793	CAP3_round1_contig_8075
Programmed cell death protein 5	Programmed cell death protein 5-like	2.59	0.00128102	Bot_rep_c35773
Tubulin α−1A chain	Tubulin α−1A chain	3.41	0.09200355	Bot_rep_c58495
Tubulin α−3C/D chain	Tubulin α−1A isoform 1 chain	7.01	0.08838786	CAP3_round1_contig_7678
Tubulin α−3E chain	Tubulin α−1A isoform 2 chain	6.58	0.05895041	454_debris_rep_c1099
Tubulin β−2C chain	Tubulin β chain like isoform 1	3.08	0.04459645	Bot_rep_c57029

To further analyze the biological roles of the 7,695 differentially expressed genes, we performed gene ontology analysis (GO) using the human genes as a proxy, resulting in 350 annotations based on Biological Processes. The 350 GO annotations revealed that in general biological processes involving but not limited to: actin cytoskeleton, metabolic processes (amino acid transport, ATP catabolic process, carbohydrate metabolic process, lipid metabolic processes, glucose metabolic process, fatty acid metabolic process), apoptotic process (induction of apoptosis), microtubule cytoskeleton organization (including microtubule-based movement), blood coagulation, branching morphogenesis of a tube, cell processes (adhesion, death, differentiation, cell division, negative regulation of cell proliferation, negative regulation of cell size), DNA metabolic process (damage response, repair, histone methylation), negative regulation of angiogenesis, oxidation-reduction process, phagocytosis engulfment and recognition, protein processes (glycosylation, homooligomerization, modification process, monoubiquitination, phosphorylation, transport, ubiquitination), regulation of muscle contraction, regulation of vascular permeability, response to oxidative stress, RNA metabolic process (splicing, catabolic process, processing), TOR signaling cascade, among others (Supplementary Table 2). Some of these GO annotations indicate genes with known roles in vascular aging, such as: actin cytoskeleton, apoptosis processes, cell redox homeostasis, DNA metabolic processes and repair, ECM organization, regulation of vascular permeability among others (Gourlay and Ayscough, 2005a; Fridlyanskaya et al., 2015; Bautista-Nino et al., 2016; Regina et al., 2016; Supplementary Table 2). Specifically, we found genes related to the following annotations: actin cytoskeleton organization, negative regulation of actin bundle assembly, cell morphogenesis involved in differentiation, cell proliferation, cellular membrane organization, cytoskeleton-dependent intracellular transport, endothelial cell differentiation, extracellular matrix organization, microtubule based process, mitosis, negative regulation of angiogenesis, regulation of cell adhesion, and negative regulation of cell size. These results are consistent with the literature suggesting that vascular aging is related to cytoskeletal changes which affect cell shape and size, as well as changes in ECM composition (Duca et al., 2016).

To confirm these analyses, we selected a subset of genes and compared gene expression in young and old vascular tissue isolated from other wild-type genotypes using quantitative reverse-transcription Polymerase Chain Reaction (qPCR). We assessed the expression of *actin* and *tubulin alpha-1A*, which are involved in regulating the cytoskeleton, as well as *collagen 1 and 2, tenascin-r* and *lysyl oxidase*, which are involved in regulating of the ECM. In all cases, these genes showed a reduction in expression in old vascular tissue as compared to the measured expression levels in young vessels (Table 2, Supplementary Figure 2).

### Cytoskeletal Alterations Are Correlated to Shape and Size Changes of Aged Vascular Cells

**Figure 3:**
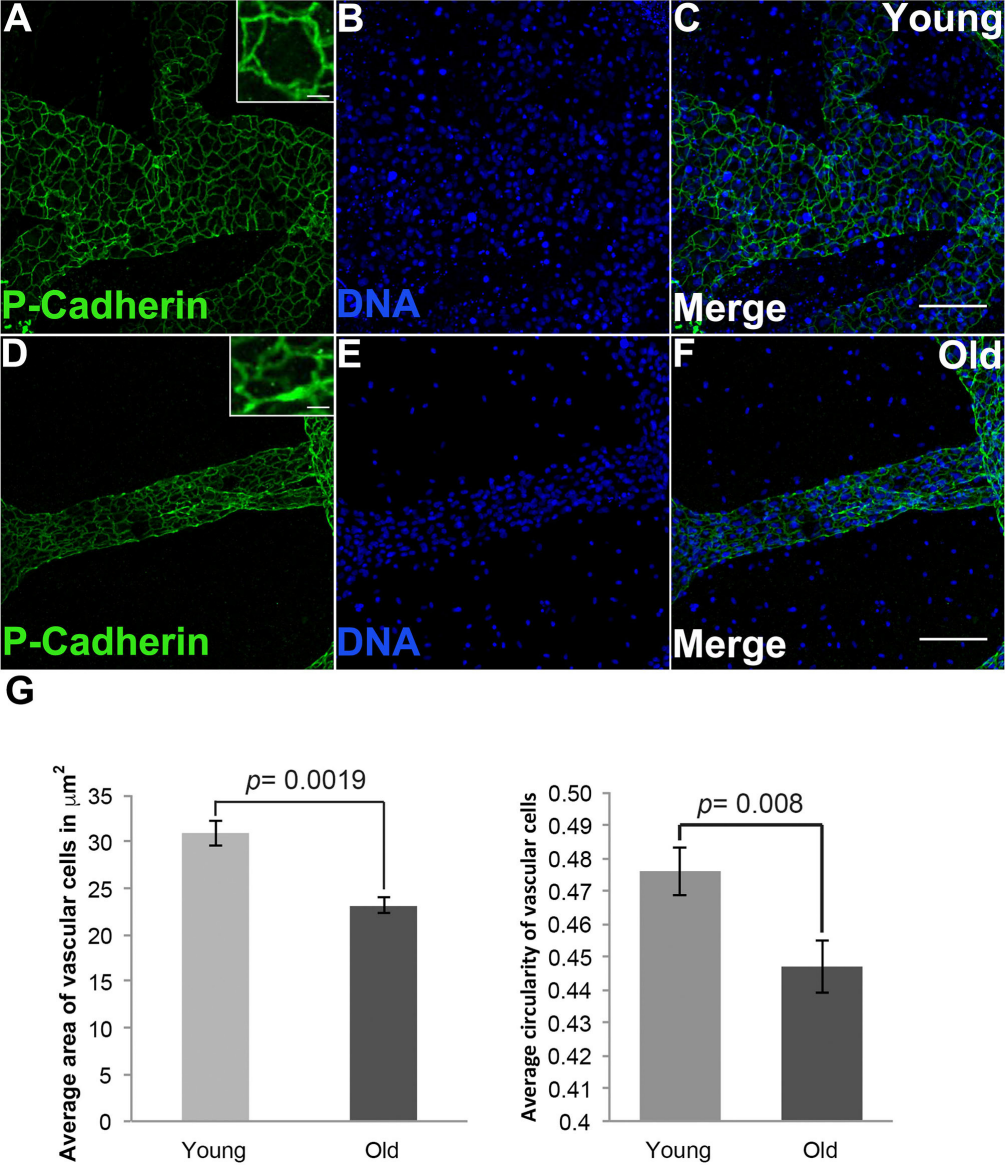
Shape and size changes of aged vascular cells. **(A–C)** Young vessels contain cells that are round on average. **(A)** Representative images showing the cell boundaries, labeled via fluorescent immunostaining of Pan-Cadherin, in the blood vessels of young *Botryllus* colonies counterstained with Hoechst 33342 to visualize the cell nucleus [**(B,C)** merged] (inset showing zoomed-in image of a single young cell). **(D–F)** Old vessels contain cells that are elongated (on the vessel long axis) on average. **(D)** Representative images showing the cell boundaries, labeled via fluorescent immunostaining of Pan-Cadherin, in the blood vessels of old *Botryllus* colonies counterstained with Hoechst 33342 [**(E,F)** merged] (inset showing zoomed-image of a single old cell). **(G)** Average area in μm^2^ and circularity (where a value of 1 indicates a perfect circle) of both young and old cells found within the vessels. For clarity only circularity values ranging from 0.4 to 0.5 are shown. Statistical analysis was performed using the Student's *t*-test. Scale bars: 50 and 5 μm for insets on **(A,D)**.

To investigate how cell size changed during vascular aging of *Botryllus* colonies we used an anti-Pan Cadherin antibody to outline the cell boundaries (Madhu et al., 2020), allowing us to quantify cell size and shape by measuring both area and circularity (where a circularity value of 1 indicates a perfect circle). Figure 3 shows representative micrographs from young and old vessels. In order to prevent bias, we sampled many regions including radial vessels and regions far away from the bodies, and we did not include ampullae. By sampling random regions of the vascular-bed we ensure that any detected changes are significant on the length scale of the vascular bed and not limited to specific regions. Cells in young vessels have an area of 30.9 ± 1.3 μm^2^; in contrast the cell size of old vessels is reduced with an area of 23.1 ± 0.86 μm^2^ (significant at *p* = 0.0019, two-tailed *t*-test). Cells in young vessels have a circularity of 0.47 ± 0.01; in contrast cells in old vessels have a circularity of 0.44 ± 0.01 (significant at *p* = 0.008, two-tailed *t*-test), indicating that the cell shape is slightly more elongated (Figure 3G). This is consistent with the observed shapes of the cells within each vessel type (compare Figures 3A,D inset).

**Figure 4:**
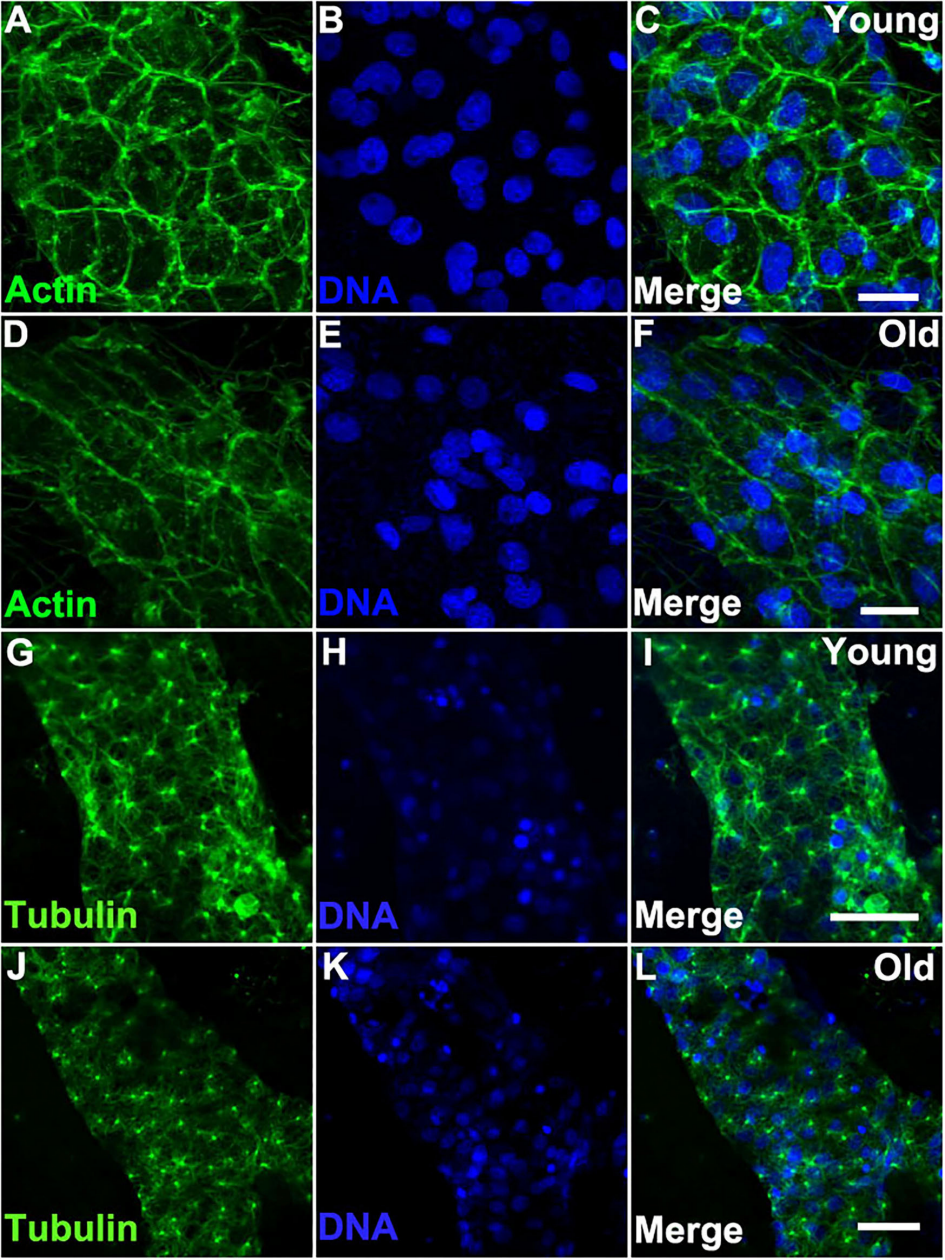
Cytoskeleton changes in aged vascular cells. **(A–C)** Young vessels exhibit large polygonal round cells. **(A)** Representative images of the fluorescent actin stained with phalloidin-594 (green) in vessels of young *Botryllus* colonies which were counterstained with Hoechst 33342 to visualize the cell nuclei [**(B,C)** merged]. **(D–F)** Old vessels exhibit stretched polygonal cells. **(D)** Representative images of the fluorescent actin stained with phalloidin-594 (green) in vessels of old *Botryllus* colonies which were counterstained with Hoechst 33342 to visualize the cell nuclei [**(E,F)** merged]. **(G,H)** Young vessels exhibit abundant arrays of microtubules. **(G)** Representative fluorescent immunostaining of α-Tubulin vessels of young *Botryllus* colonies counterstained with Hoechst 33342 [**(H,I)** merged]. **(J–L)** Old vessels exhibit less abundant arrays of microtubules. **(J)** Representative fluorescent immunostaining of α-Tubulin vessels of old *Botryllus* colonies counterstained with Hoechst 33342 [**(K,L)** merged]. Scale bars: 50 μm.

We reasoned that the morphological changes observed in older vascular cells may be induced by changes in the cytoskeleton. Indeed, we found that the actin cytoskeleton of old vessels is stretched and disorganized when compared to that of young vessels (Figures 4A–F). In contrast, when we compared the actin cytoskeleton within the zooid bodies, no differences were observed as a function of organism age (Supplementary Figure 3). Additionally, immunostaining of α-Tubulin (using an antibody that cross-reacts with multiple species) revealed that bundles of microtubules in young vascular cells are denser and contain longer microtubules as compared to the cells of old vessels (Figures 4G–L).

### ECM Alterations Are Related to the Functional Decline of Aged Vascular Cells

**Figure 5:**
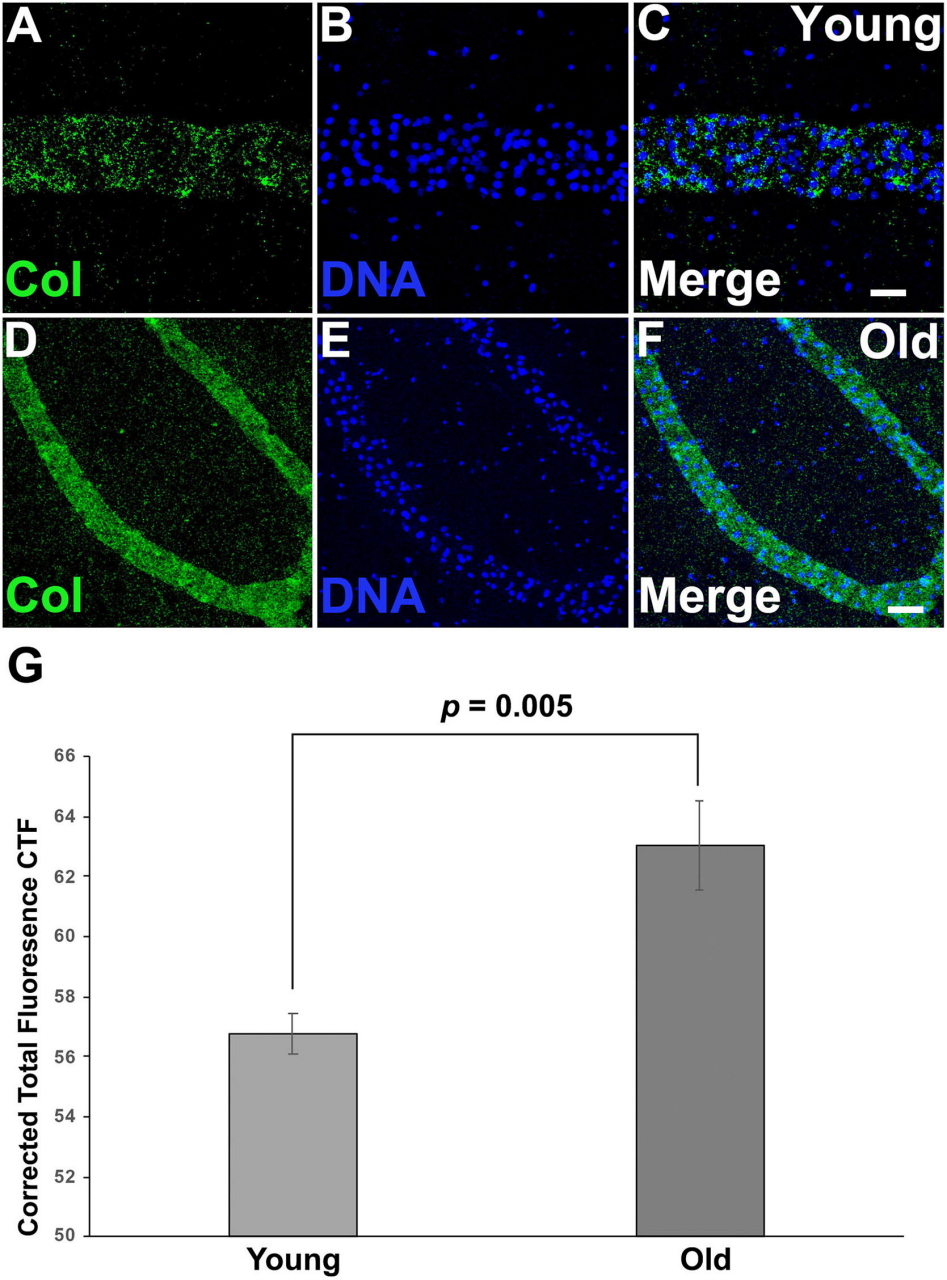
Collagen is accumulated on aged vascular cells. **(A–C)** Young vessels show a sparse, punctate positive signal for putative Collagen immunostaining. **(A)** Representative image showing the fluorescent immunostaining of putative Collagen vessels of young vessels counterstained with Hoechst 33342 [**(B,C)** merged]. **(D–F)** Old vessels show a strong accumulation of putative Collagen. **(D)** Representative fluorescent immunostaining of putative Collagen vessels of old vessels counterstained with Hoechst 33342 [**(E,F)** merged]. **(G)** Corrected Total Fluorescence (arbitrary units) quantification of young vs old vessels. Statistical analysis was performed using the Student's *t*-test. Scale bars: 10 μm.

Both differential expression and GO analysis indicated that expression of genes related to the ECM changed as colonies aged (Supplementary Tables 1, 2). Specifically, several types of collagens were found to be differentially expressed between young and old colonies (Supplementary Table 1). Immunostaining using an anti-chicken collagen II gave a staining in *Botryllus* samples for a putative tunicate collagen (referred to as collagen within) (Figures 5A–F). This antibody cross reacts with many vertebrate species and this is the first report of immunostaining on an invertebrate, it must be noted that a western blot did not detect the denatured protein. As a negative control, we labeled with a secondary antibody only (Supplementary Figures 4G–L) for both young and old, and found no measurable signal. These studies suggest that collagen is heavily accumulated in aged vascular tissues (Figure 5), showing a stronger signal in old tissue as compared to a weak and spotty staining signal of collagen in young tissue (Figures 5A–F, Supplementary Figures 4A–F). In both cases the detected signal was exclusively localized to blood vessels. From this, we conclude that the spotty distribution seen in young vessels is reliable. We quantified the fluorescence signal from the immunostained micrographs and calculated the corrected total fluorescence (CTF). The difference in signal intensity is significant (young CTF = 56.7 ± 0.7 and old CTF = 63 ± 1.5, *p* = 0.005, arbitrary units). With this, we confirmed that there is a significant accumulation of collagen on old vessels when compared to young ones.

### Inducible Vascular Regression Declines With Age

**Figure 6:**
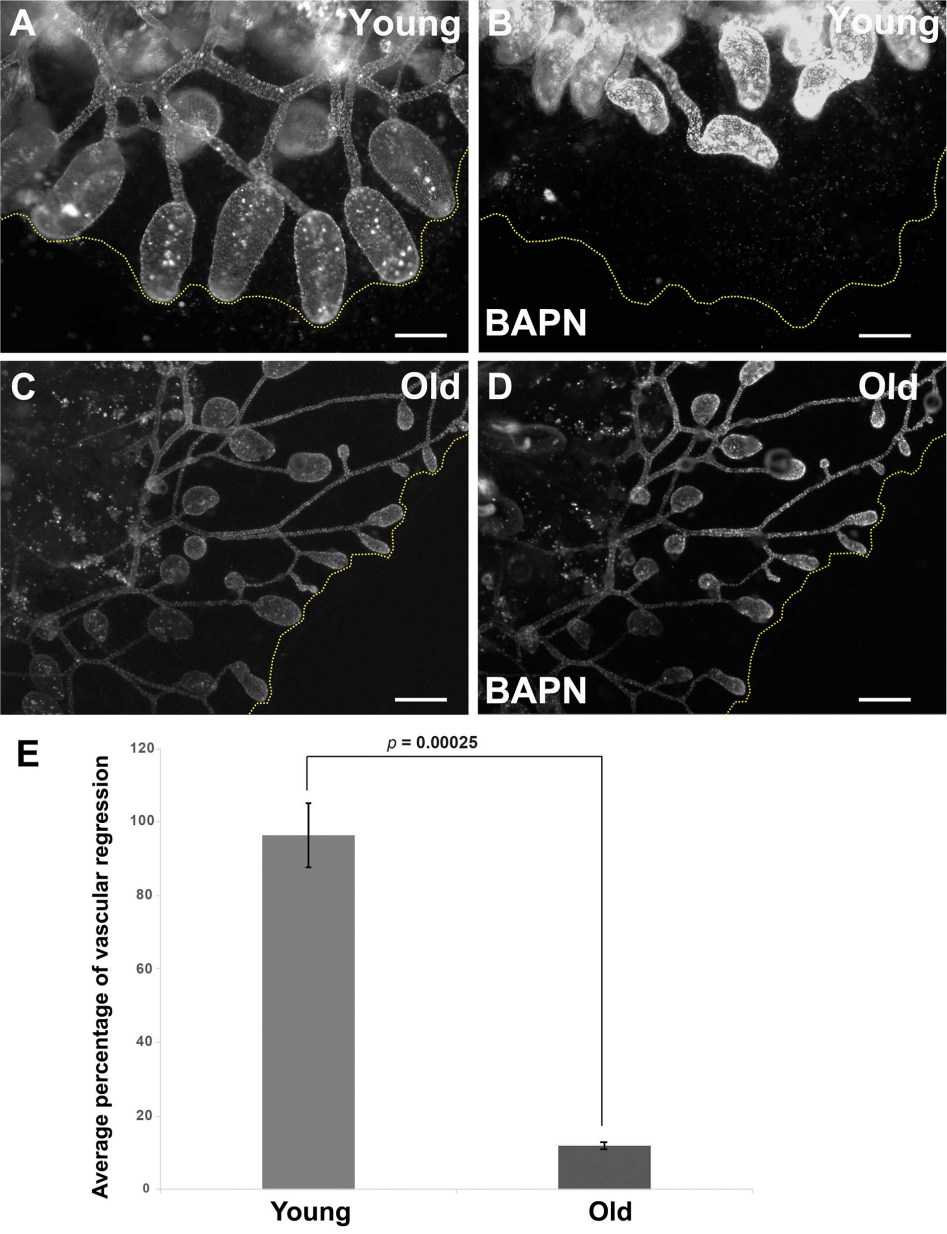
Inducible vascular regression declines with age. **(A,B)** Representative fluorescent micrographs of the vascular bed labeled with BSA-Alexa Fluor 594 of a young colony before **(A)** and after BAPN treatment **(B)**. **(C,D)** Representative fluorescent micrographs of the vascular bed labeled with BSA-Alexa Fluor 594 of an old colony before **(C)** and after BAPN treatment **(D)**. **(E)** Average percentage changes of vascular bed perimeter were assessed 16 h following BAPN exposure of young and old *Botryllus* colonies. Statistical analysis was performed using the Student's *t*-test. Scale bars: 1 mm.

We next assessed the impact of these age-dependent changes in ECM properties on tissue-level function through analysis of induced regression of young and old vasculature. Lysyl oxidase (Lox) is an extracellular enzyme that is involved in the cross-linking of collagen molecules into fibrils. We have previously shown (Rodriguez et al., 2017; Madhu et al., 2020) that the vasculature of young *Botryllus* animals rapidly and globally regresses in response to Lox inhibition using the small molecule inhibitor, BAPN. BAPN treatment changes the structure of the vascular basement membrane in as little as 16 h, with collagen fibrils unwinding and becoming disordered. The vasculature responds to this change in structure by undergoing a global regression, due to the extrusion and apoptosis of a subset of vascular cells (Rodriguez et al., 2017). Interestingly, Lox does not enzymatically cross-link collagen molecules, rather it modifies amino acid side chains of collagen proteins, thereby allowing them to spontaneously form cross-links. Thus, the rapid change in collagen fibril structure due to Lox inhibition suggests that the collagen fibrils are very dynamic, and in a constant state of disassembly and reformation (Rodriguez et al., 2017). In this study, we found that the magnitude of BAPN-induced vascular regression is significantly reduced in old animals (Figure 6). Exposing young animals (<4 months old) to BAPN results in large regression of the extracorporeal vessels (Figures 6A,B,E, Supplementary Video 3) with a 96.4% decrease in the size of their original vascular bed (*n* = 10), as previously observed (Rodriguez et al., 2017). In contrast, old animals (>1-year-old) show a significantly reduced response to this treatment (Figures 6C–E, Supplementary Video 4) with only 11.8% decrease in the size of their original vascular bed (*n* = 10). This roughly 8-fold-decrease in response to Lox inhibition correlates with a drop of *lox* expression in old vascular tissue (Supplementary Figure 2B).

### Cell Shape and Size Is Maintained in Regenerated Aged Vascular Cells

**Figure 7:**
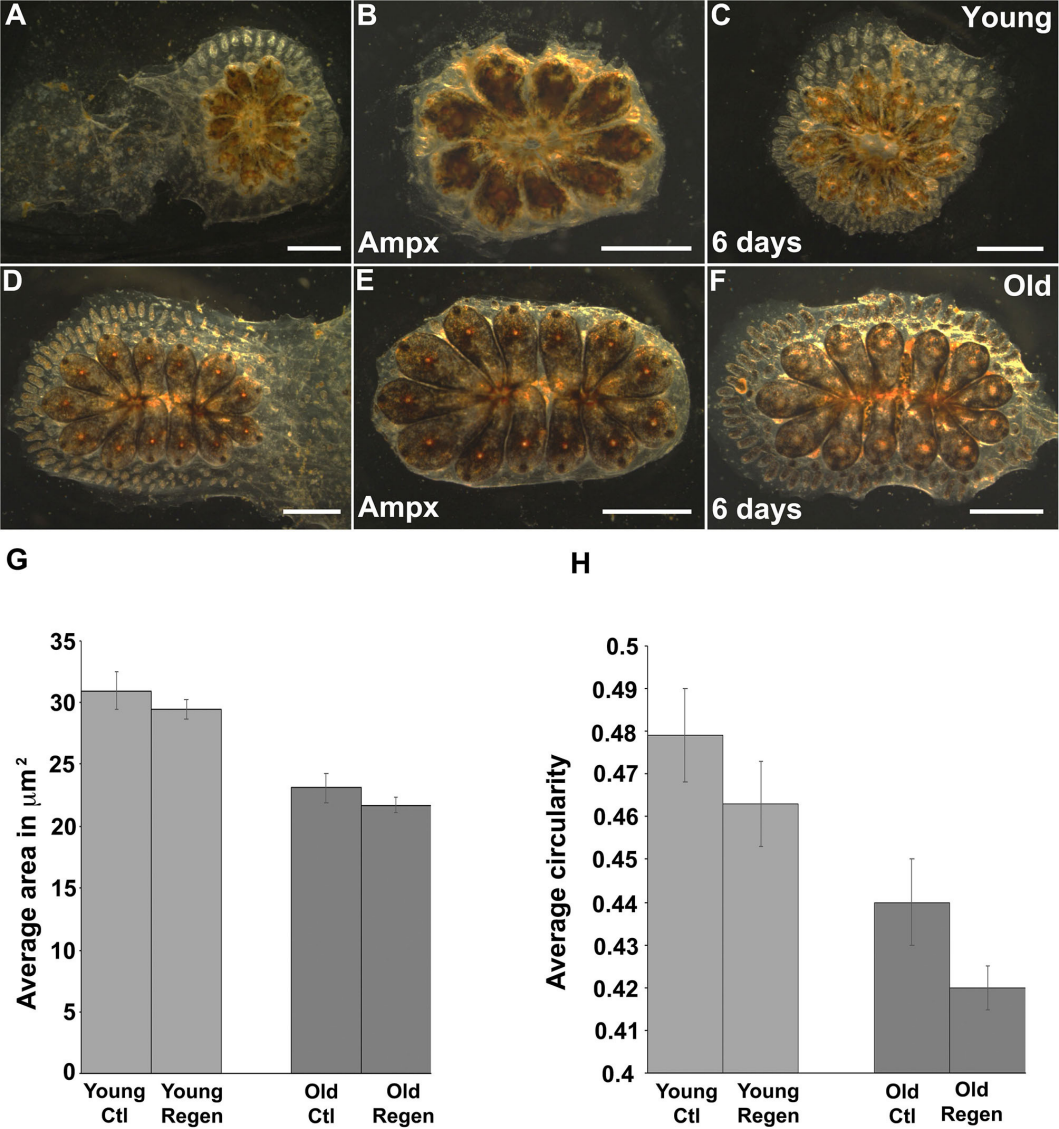
Cytoskeleton changes on aged vascular cells retained the same phenotype upon regeneration. **(A–C)** Young colony before and after ampullectomy and fully regenerated 6 days later. **(A)** Representative bright field images of young colony before surgery. **(B)** Bright field image of a young colony immediately following ampullectomy (Ampx) and **(C)** Bright field image of a young colony 6 days post-ampullectomy with fully regenerated vessels. **(D–F)** Old colony before and after ampullectomy and fully regenerated 6 days later. **(D)** Representative bright field images of old colony before surgery. **(E)** Bright field image of an old colony immediately following ampullectomy and **(F)** Bright field image of an old colony 6 days post ampullectomy with fully regenerated vessels. **(G,H)** Regenerated blood vessels were stained with Pan-Cadherin and the **(G)** average area and **(H)** circularity of cells in control (Ctl) and regenerated (Regen) tissue for both young and old animals. Statistical analysis was performed using the Student's *t*-test. Scale bars: 2 mm.

Most organisms display a progressive decline in their regenerative capabilities with age, including a reduction in tissue turnover and inability to replace lost or damaged cells (Yun, 2015; Seim et al., 2016). *Botryllus* blood vessels are an excellent model for studies of regeneration: after surgical removal of the ampullae and marginal vasculature the animals fully regenerate their vessel bed in ~1 week (Gasparini et al., 2008; Tiozzo et al., 2008; Braden et al., 2014). To investigate if the regenerative capability of *Botryllus* is compromised with age, we surgically removed most of the peripheral ampullae and marginal blood vessels (portions of the vasculature cannot be removed because they are located underneath the zooids) on both young and old colonies and compared their regeneration ability and rate. Interestingly, we did not observe any differences with age: in both cases, the animals fully regenerated and it took about 6 days to extend the new vessel bed (Figures 7A–F).

We hypothesized that the newly-formed blood vessels would acquire a young phenotype as vascular cells underwent cell division to enable vessel regeneration. To investigate this experimentally, we fixed and immunostained with anti-Pan-Cadherin both young and old colonies 6 days post-surgery and performed the same morphometrics analysis as described above. Surprisingly, we found newly regenerated vascular cells retain a similar phenotype as their parental cells—suggesting that the age-dependent phenotype is heritable. In detail, in young animals, vascular cells have an area of 30.9 ± 1.3 μm^2^ and 29.4 ± 1.3 μm^2^ and a circularity of 0.47 ± 0.07 and 0.46 ± 0.01 in control and regenerated tissue, respectively (Figures 7G,H, Supplementary Figures 5A,B). Similarly, in old animals, vascular cells have an area of 23.1 ± 0.8 μm^2^ and 21.7 ± 1.25 μm^2^, and a circularity of 0.44 ± 0.01 and 0.42 ± 0.01 in control and regenerated tissue, respectively (Figures 7G,H, Supplementary Figures 5C,D). While the differences in size and shape between control and regenerated cells are significant for both young and old vessels (Supplementary Figure 5), the mean values of both the area and circularity much more closely resemble the parental age phenotype as compared to the regenerated cells derived from young vs. old parental tissues.

## Discussion

Here we investigate a unique *in vivo* model of vascular aging, the colonial invertebrate chordate, *Botryllus schlosseri*. We demonstrate that over time, aging vascular cells undergo distinct and consistent morphological changes that are inherited by newly formed vascular cells upon growth of new vasculature, either normally, or following stimulation by surgical ablation. Importantly, this aging process is consistent among diverse genotypes. We establish that in aging colonies, the multiple branching of blood vessels leads to smaller diameter vessels that have a direct effect on blood flow and the amount of blood cells in circulation. We identify age-dependent changes in the expression profile of both cytoskeletal and ECM genes, and show that these changes correlate with morphological changes of vascular cells, and are consistent with functional changes as assessed by inducible vascular regression.

A key result of this study is the finding that while the ability of the vascular tissue to regenerate is not age-dependent, cells within the newly formed vessels retain the age phenotype of the derivative tissue: young animals regenerate young vascular cells, and old genotypes regenerate old. We have also found that, similar to studies in mice, vascular regeneration is not dependent on the presence of either mobile or resident vascular progenitors: the majority of cells are usually quiescent, and new vessel growth is due to a subset of resident vascular cells that re-enter the cell cycle during homeostatic or regenerative angiogenesis (Braden et al., 2014; McDonald et al., 2018). The lack of an adult vascular stem cell in either species is not surprising, as over time an injury could occur that requires new vessel growth at any place in the body, thus the entire vasculature must be responsive to regenerative stimulus. What is surprising is that the vascular regeneration we are studying occurs after a ablation of the peripheral vascular bed (Figure 7), and includes regeneration of the entire vessel: cells, basement membrane and tunic. In summary, the aging phenotype is not just heritable, it is global.

It is intriguing that in both cases, new vessel growth is not dependent on the presence of progenitor cells, and the majority of vascular cells are usually quiescent (Braden et al., 2014; McDonald et al., 2018). In contrast, many mammalian tissues are in a constant state of regeneration, which is fueled by tissue specific stem cells. Aging in these tissues is thought to be due to damage and accumulation of mutations within, often followed by competition between, the corresponding stem cells, which in turn affect the differentiated progeny (Hayflick, 1998; Beerman et al., 2010; Sousounis et al., 2014; Jaiswal and Ebert, 2019; Liu et al., 2019). So how do vascular cells age? While the mechanisms are not clear, here we show that in *Botryllus* there are global, heritable, age-dependent changes in typically quiescent vascular cells that affect both the structure and function of the vessels, similar to mammals. The characteristic progression of vascular aging in *Botryllus* will allow us to characterize global epigenetic and gene expression changes that underlie these heritable aging phenotypes.

As aging is associated with significant changes in gene expression, understanding these changes over the lifespan of an organism is essential to gain new insights into the molecular processes underlying cellular and organismal aging (Nakamura et al., 1999; Prall et al., 2007; de Magalhaes et al., 2009). In the present study, we examined age-related changes in gene expression and found many processes in common with mammals. In humans, age-related molecular changes include transcriptional and epigenetic modifications that directly influence the composition of the ECM, which in turn alters the cytoskeleton causing a number of age-related diseases and dysfunctions (Phillip et al., 2015). The differentially expressed genes we identified by this approach also led us to investigate changes in the actin cytoskeleton in aging vascular cells. We show that the actin cytoskeleton of aged vascular cells is significantly altered: the actin fibers are stretched, elongated and disorganized when compared to young vascular cells which exhibit a polygonal shape and well-organized actin cytoskeleton. Aged vascular cells have an altered cytoskeleton and altered overall morphology when compared to young vascular cells. This corresponds to studies in human and model organisms where a number of genes associated with longevity are involved in the regulation of endothelial cell functions (Schachter et al., 1994; Willcox et al., 2008; Li et al., 2009; Zhao et al., 2012; Soerensen et al., 2013).

Interestingly, prior studies of yeast (Gourlay and Ayscough, 2005c) suggest that the actin cytoskeleton is directly involved in the regulation of aging; additionally, aged human fibroblasts are characterized by a disordered actin cytoskeleton that in turns negatively affects cell mobility and contractility (Reed et al., 2001). In both tissue culture cells and yeast a decrease in actin turnover results in large aggregates of F-Actin which in turn induces apoptosis (Posey and Bierer, 1999; Odaka et al., 2000; Gourlay et al., 2004; Gourlay and Ayscough, 2005b). In support of this correlation, a yeast mutant with increased actin turnover exhibited an increased lifespan (Goodman et al., 2003; Winder et al., 2003; Gourlay et al., 2004).

Both gene expression and immunostaining using an anti-collagen antibody from chicken suggest that the basement membrane of the vascular cells in *Botryllus* significantly accumulates collagen as the cells age. Moreover, these ECM changes correlate with the ability of vascular cells to respond to inhibition of collagen crosslinking. We previously showed that pharmacological inhibition of Lox, which is highly expressed by vascular cells, can trigger vascular regression in young animals of *Botryllus*, due to the disruption of collagen fibers in the lumen of the blood vessels, which in turn triggers anoikis and extrusion of vascular cells (Rodriguez et al., 2017). Here, we show that this induced regression is substantially suppressed in old animals. LOX does not reversibly crosslink collagen, rather it modifies the lysine side chains, which subsequently bind non-enzymatically. Electron microscopic analyses showed a clear unwinding of fibrils in LOX treated animals (Rodriguez et al., 2017), suggesting that in young colonies fibrils are very dynamic, being constantly created and destroyed. Our results suggest that changes in the turnover rate of collagen fibrils may slow with age, consistent with the accumulation of fibrils we observed in older colonies.

While we do not know what proteins are responsible for fibril dissociation, an age dependent change in the kinetics of dynamic remodeling could be responsible for changes in the ECM structure, and an increase in the overall crosslinking content, rendering the ECM stiffer and more mechanically stable over time. A similar phenotype is observed in human arteries, where collagen molecules accumulate cross-links with age, altering their structural and functional properties (Aronson, 2003). Moreover, age associated changes of the cytoskeleton and ECM in endothelial cells have also been observed in mice (Fleenor et al., 2012). The aged cell phenotype we observe in *Botryllus* also resembles that of vascular smooth muscle cells by highly expressing alpha actin (ACTN1) and collagen I (COL1A1) which may contribute to vessel stiffening (Urry, 1971; Urry and Onishi, 1974; Otto et al., 1999; Fleenor et al., 2010, 2012; Kohn et al., 2015).

In our observations of vascular blood flow, we observe that old vessels have significantly smaller diameters than young vessels. We also find that blood flow velocity depends on vessel diameter in a manner entirely consistent with Murray's Law for branched vascular networks (Murray, 1926), indicating that the *Botryllus* vasculature follows similar evolutionary pressures for transport optimization as other systems known from the literature (Yap et al., 2014; Konrad, 2016). While we do observe a net reduction of flow velocities in older vessels compared to young ones, these differences are consistent with the predictions of Murray's Law for the observed reduced diameters in old vessels. Thus, the evidence suggests that the effective changes in blood flow during aging are caused primarily by the progressive branching behavior of the vasculature during aging—i.e., the vasculature evolving toward a more branched morphology with increasingly more and narrower blood vessels, which have an accordingly slower blood flow velocity—as opposed to macroscopic mechanical changes to the vasculature, or systematic changes in blood pressure. It is possible that this unidirectional branching progression of the vasculature follows the organism's evolving needs (i.e., a more highly branched network with slower blood flow may be more efficient in supplying the aging organism). On the other hand, the progressively branching network morphology may reach an efficiency threshold that represents an endpoint of the aging process that triggers death–either when the blood flow slows down so much that adhesive forces become more relevant, so that blood and pigment cells start sticking to the vessel walls and impede blood flow; or when the blood vessels become so narrow that they mechanically collapse.

In summary, here we show that age-related changes in the cytoskeleton and the extracellular matrix reshape vascular cells into an elongated form that is accompanied by the highly likely accumulation of collagen, and these changes are consistent among diverse wild-type genotypes, and resemble changes that occur in mammalian vessels during aging. Interestingly, the young or aged phenotype of both the cells and the extracellular matrix are heritable and, and maintained in daughter cells during new vascular growth, suggesting that epigenetic changes underlie aging. The global nature and progression of aging in *Botryllus* represents a new and powerful model to study vascular aging.

## Data Availability Statement

The datasets presented in this study can be found in online repositories. The names of the repository/repositories and accession number(s) can be found in the article/Supplementary Material.

## Author Contributions

DR study conception and design, acquisition of data, analysis and interpretation of data, drafting of manuscript, critical revision of the manuscript, and final approval of the manuscript. DT, SK, and RM acquisition of data, analysis and interpretation of data, critical revision of the manuscript, and final approval of the manuscript. AD, MV, and DL study conception and design, analysis and interpretation of data, drafting of manuscript, critical revision of the manuscript, and final approval of the manuscript. All authors contributed to the article and approved the submitted version.

## Conflict of Interest

The authors declare that the research was conducted in the absence of any commercial or financial relationships that could be construed as a potential conflict of interest.

## Supplementary Material

Supplementary Figure 1 **(A)** Minimal projection image of a blood vessel. The image also shows the center line segment (blue) used for generating the kymograph, and the line segment (dashed red line) for measuring diameter of the vessel. **(B,C)** Sample trace of the (time-resolved) flow velocity measured through autocorrelation analysis. **(B)** Young animal (same as kymograph in Figure 2A); **(C)** old animal (same as kymograph in Figure 2B). **(D)** Scatter plot of volumetric flow rate as a function of cross-sectional area of the blood vessels from young and old animals. **(E)** Mean flow velocity in blood vessels within the 25–35 μm range in young and old animals. **(F)** Scatter plot of volumetric flow rate as a function of diameter of the blood vessels from young and old animals. Gray curve is the result of fitting the data to a power law.

Supplementary Figure 2 Vascular tissue changes expression of cytoskeletal and ECM genes with age. Surgically removed extracorporeal vasculature of young and old colonies was used to compare the relative expression ratio of both cytoskeleton and ECM genes normalized to young vascular tissue. **(A)** qPCR analysis actin and tubulin for young and old vascular tissue shown as fold change normalized to young vascular tissue. **(B)** qPCR analysis collagen-1 and 2, Lox and Tenascin-r for young and old vascular tissue shown as fold change normalized to young vascular tissue.

Supplementary Figure 3 The cytoskeleton of zooids does not change with age. **(A–C)** Zooid from a young colony. **(A)** Representative fluorescent actin staining with phalloidin-594 (green) of a zooid of a young *Botryllus* colonies counterstained with Hoechst 33342 [**(B,C)** merged]. **(D–F)** Zooid from an old colony. **(D)** Representative fluorescent actin staining with phalloidin-594 (green) of a zooid of a young *Botryllus* colonies counterstained with Hoechst 33342 [**(E,F)** merged]. Scale bars: 200 μm.

Supplementary Figure 4 Collagen is accumulated on aged vascular cells. **(A–C)** Young vessels show a sparse, punctate positive signal for putative Collagen immunostaining. **(A)** Representative image showing the fluorescent immunostaining of putative Collagen vessels of young vessels counterstained with Hoechst 33342 [**(B,C)** merged]. **(D–F)** Old vessels show a strong accumulation of putative Collagen. **(D)** Representative fluorescent immunostaining of putative Collagen vessels of old vessels counterstained with Hoechst 33342 [**(E,F)** merged]. **(G–I)** Young vessels immunostained with secondary antibody only as a negative control, and counterstained with Hoechst 33342 [**(H,I)** merged]. **(J–L)** Old vessels immunostained with secondary antibody only as a negative control counterstained with Hoechst 33342 [**(K,L)** merged]. Scale bars: 10 μm.

Supplementary Figure 5 Cytoskeletal changes in aged vascular cells are retained upon regeneration. **(A–C)** Regenerated blood vessel were stained with P-Cadherin and the average area **(B,D)** and circularity **(A,C)** of cells in intact and regenerated tissue for both young **(A,B)** and old **(C,D)** animals. Despite significant differences the regenerated cells as compared to their parent control, the mean values of both circularity and perimeter much more closely resemble those of the parental age phenotype before surgery than the regenerated cells of the young and old animals resemble each other. Statistical analysis was performed using Student's *t*-test.

Supplementary Table 1 Differential expression analysis between old and young *Botryllus* colonies and gene homologs of *Homo sapiens* and *Ciona intestinalis*, base mean, base mean for old (3 years), base mean for young (4 months) fold changes, log_2_ Fold Changes, *p*-values and adjusted *p*-values included.

Supplementary Table 2 Gene Ontology Annotations based on Biological Processes (BP) Including Support, Reference support, Reference size, Hypergemetric test, Hypergemetric test corrected, and Genes Symbols.

Supplementary Video 1 Blood flow in young blood vessels. Time-lapse video of blood vessels from a young colony showing blood flow for 1 min. The images were taken every second for a period of 1 min.

Supplementary Video 2 Blood flow in old blood vessels. Time-lapse video of blood vessels from an old colony showing blood flow for 1 min. The images were taken every second for a period of 1 min.

Supplementary Video 3 Induced vascular regression of young fluorescently labeled blood vessels. Time-lapse video of a subset of blood vessels from a young colony injected with BSA-488 (which labels vascular cells), the colony was incubated in filtered seawater containing the BAPN (400 μM) for 16 h. The images were taken every 10 min for a period of 16 h. The images were taken every 10 min for a period of 16 h and compressed here to 6 s.

Supplementary Video 4 Induced vascular regression of old fluorescently labeled blood vessels. Time-lapse video of a subset of blood vessels from an old colony injected with BSA-488 (which labels vascular cells), the colony was incubated in seawater containing the BAPN (400 μM) for 16 h. The images were taken every 10 min for a period of 16 h. The images were taken every 10 min for a period of 16 h and compressed here to 6 s.
