# Supplementary figures and images for: Vascular Aging in the Invertebrate Chordate, Botryllus schlosseri
Source: Front Mol Biosci. 2021 Apr 8;8:626827. doi: 10.3389/fmolb.2021.626827 (PMC8060491; doi:10.3389/fmolb.2021.626827)

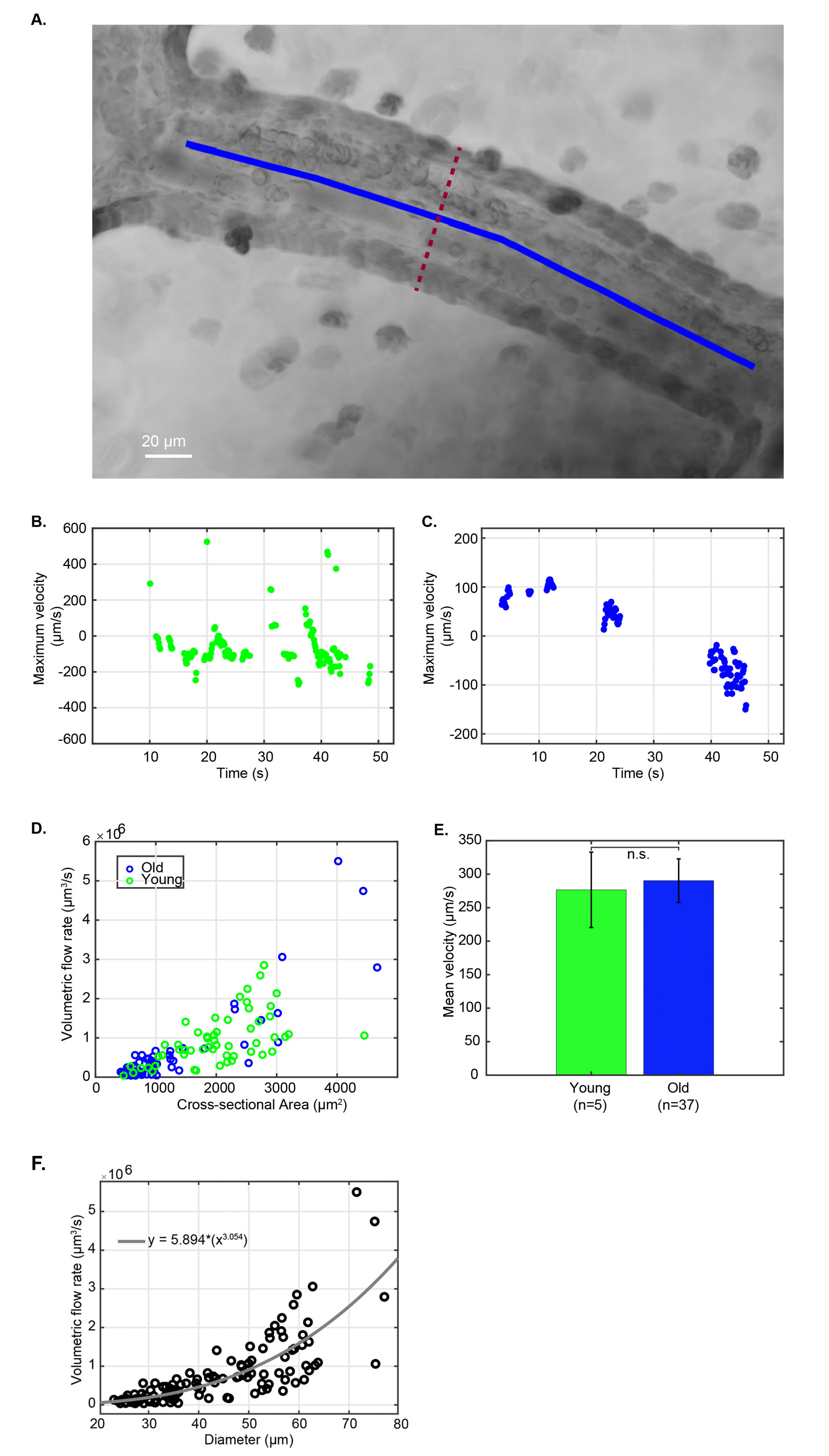

Supplement: Supplementary Figure 1 — (A) Minimal projection image of a blood vessel. The image also shows the center line segment (blue) used for generating the kymograph, and the line segment (dashed red line) for measuring diameter of the vessel. (B,C) Sample trace of the (time-resolved) flow velocity measured through autocorrelation analysis. (B) Young animal (same as kymograph in Figure 2A); (C) old animal (same as kymograph in Figure 2B). (D) Scatter plot of volumetric flow rate as a function of cross-sectional area of the blood vessels from young and old animals. (E) Mean flow velocity in blood vessels within the 25–35 μm range in young and old animals. (F) Scatter plot of volumetric flow rate as a function of diameter of the blood vessels from young and old animals. Gray curve is the result of fitting the data to a power law. [file Image_1.JPEG]

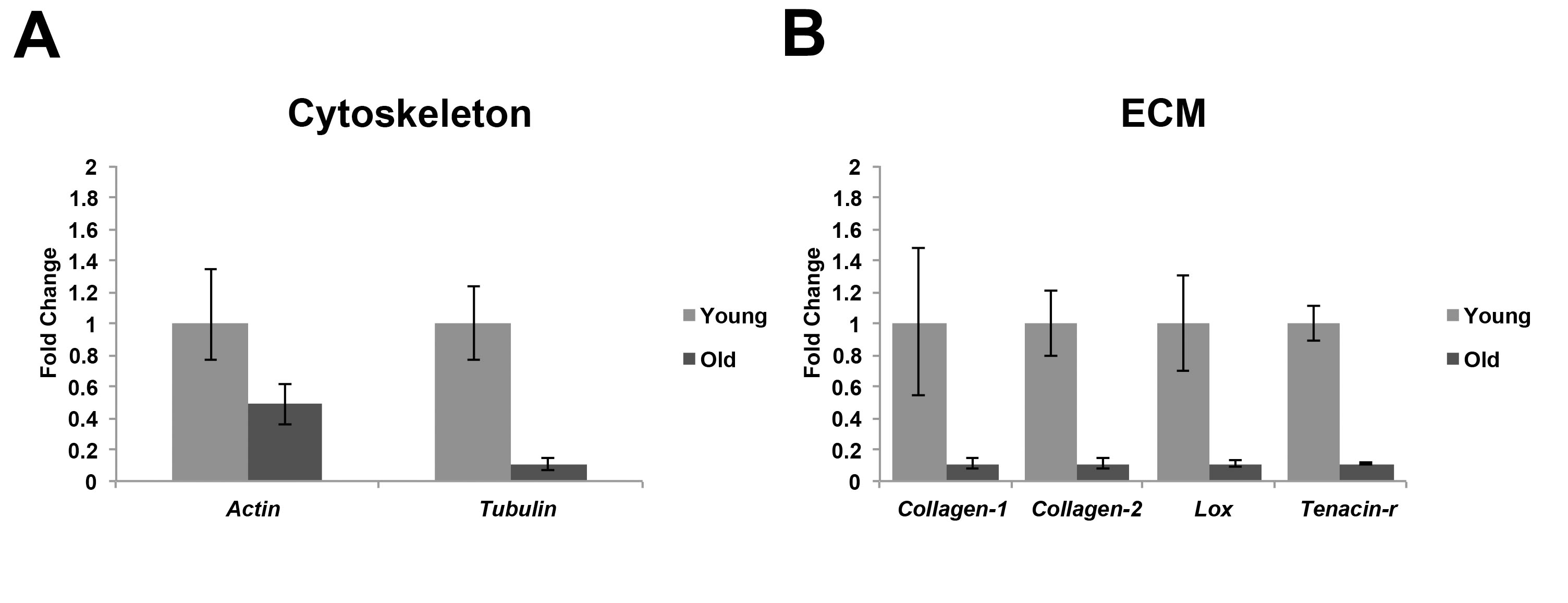

Supplement: Supplementary Figure 2 — Vascular tissue changes expression of cytoskeletal and ECM genes with age. Surgically removed extracorporeal vasculature of young and old colonies was used to compare the relative expression ratio of both cytoskeleton and ECM genes normalized to young vascular tissue. (A) qPCR analysis actin and tubulin for young and old vascular tissue shown as fold change normalized to young vascular tissue. (B) qPCR analysis collagen-1 and 2, Lox and Tenascin-r for young and old vascular tissue shown as fold change normalized to young vascular tissue. [file Image_2.JPEG]

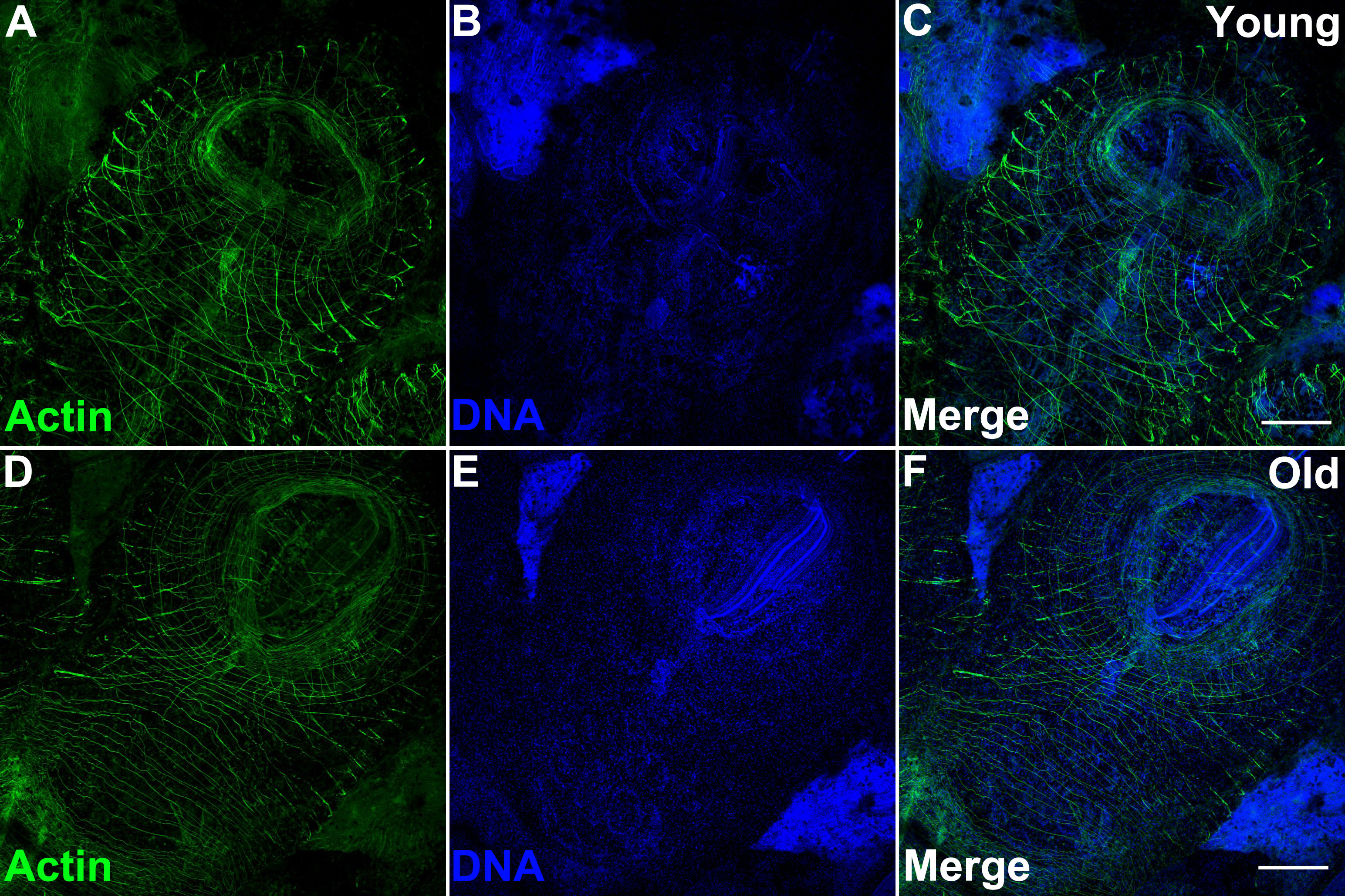

Supplement: Supplementary Figure 3 — The cytoskeleton of zooids does not change with age. (A–C) Zooid from a young colony. (A) Representative fluorescent actin staining with phalloidin-594 (green) of a zooid of a young Botryllus colonies counterstained with Hoechst 33342 [(B,C) merged]. (D–F) Zooid from an old colony. (D) Representative fluorescent actin staining with phalloidin-594 (green) of a zooid of a young Botryllus colonies counterstained with Hoechst 33342 [(E,F) merged]. Scale bars: 200 μm. [file Image_3.JPEG]

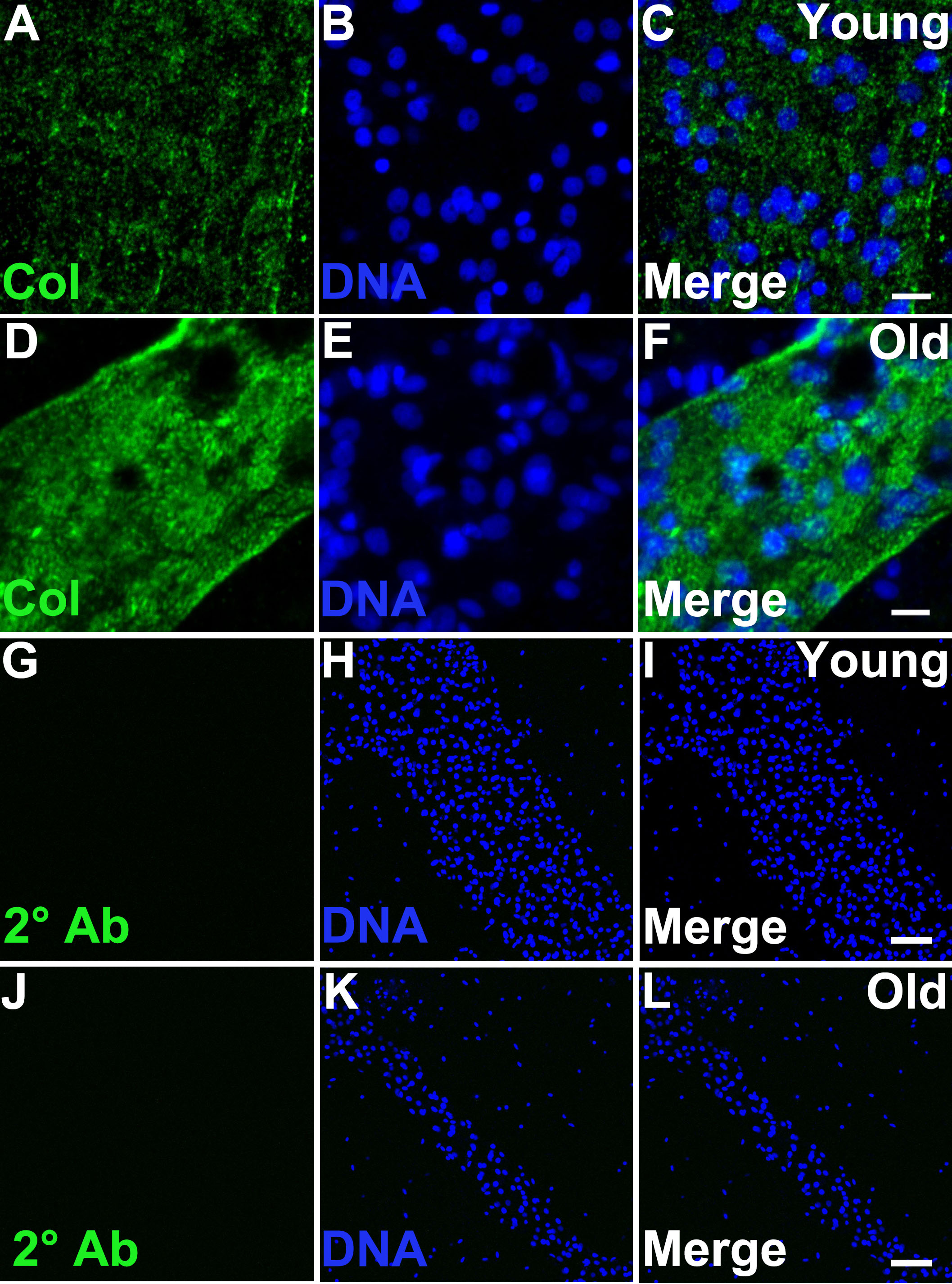

Supplement: Supplementary Figure 4 — Collagen is accumulated on aged vascular cells. (A–C) Young vessels show a sparse, punctate positive signal for putative Collagen immunostaining. (A) Representative image showing the fluorescent immunostaining of putative Collagen vessels of young vessels counterstained with Hoechst 33342 [(B,C) merged]. (D–F) Old vessels show a strong accumulation of putative Collagen. (D) Representative fluorescent immunostaining of putative Collagen vessels of old vessels counterstained with Hoechst 33342 [(E,F) merged]. (G–I) Young vessels immunostained with secondary antibody only as a negative control, and counterstained with Hoechst 33342 [(H,I) merged]. (J–L) Old vessels immunostained with secondary antibody only as a negative control counterstained with Hoechst 33342 [(K,L) merged]. Scale bars: 10 μm. [file Image_4.JPEG]

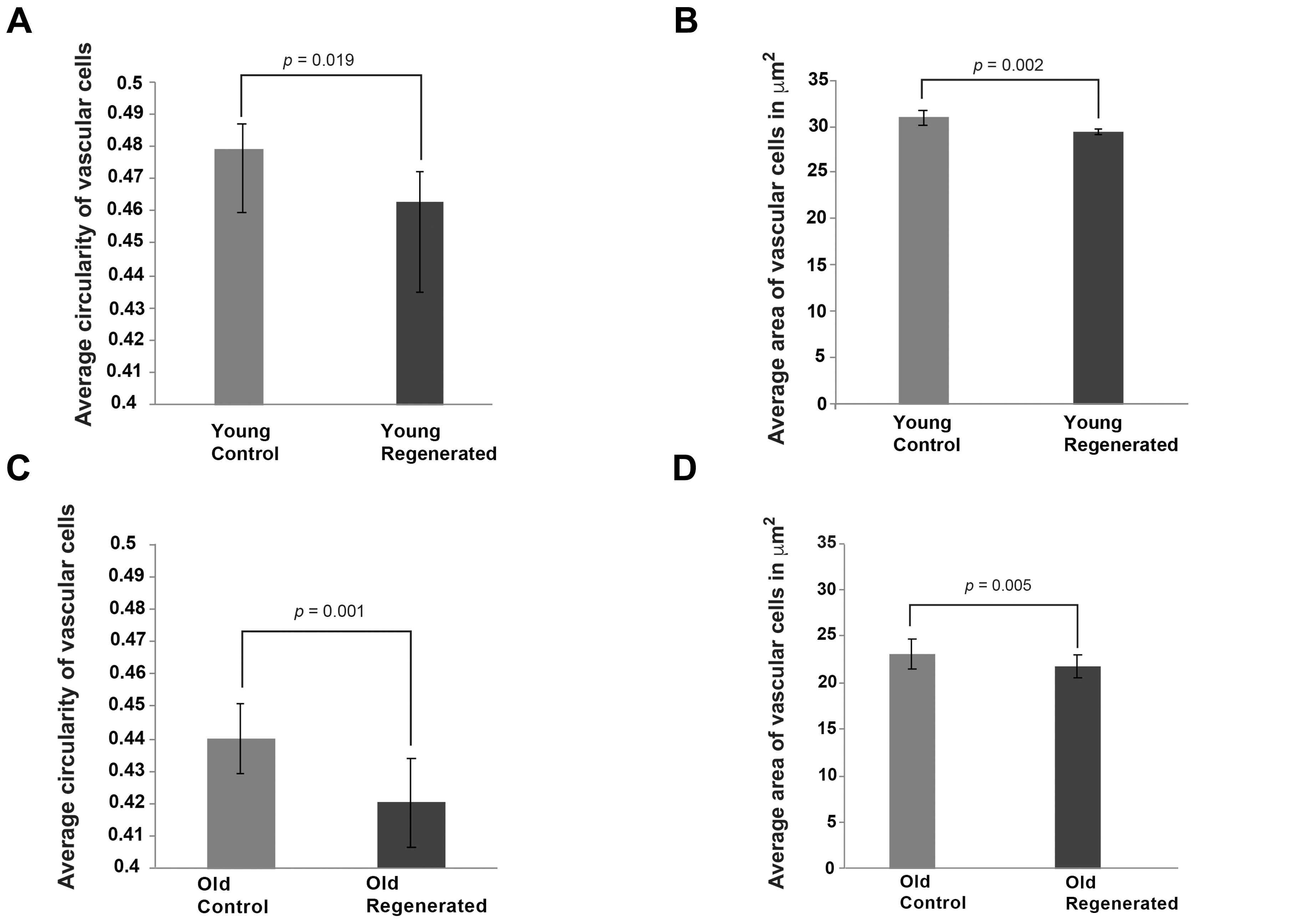

Supplement: Supplementary Figure 5 — Cytoskeletal changes in aged vascular cells are retained upon regeneration. (A–C) Regenerated blood vessel were stained with P-Cadherin and the average area (B,D) and circularity (A,C) of cells in intact and regenerated tissue for both young (A,B) and old (C,D) animals. Despite significant differences the regenerated cells as compared to their parent control, the mean values of both circularity and perimeter much more closely resemble those of the parental age phenotype before surgery than the regenerated cells of the young and old animals resemble each other. Statistical analysis was performed using Student's t-test. [file Image_5.JPEG]
